# The COVID-19 exams fiasco across the UK: four nations and two windows of opportunity

**DOI:** 10.1057/s41293-021-00162-y

**Published:** 2021-02-19

**Authors:** Sean Kippin, Paul Cairney

**Affiliations:** University of Stirling, Stirling, FK9 4LA UK

**Keywords:** COVID-19, Education policy, UK and devolved government, Multiple streams analysis, Heresthetic, Policy learning and transfer

## Abstract

All four UK and devolved governments performed a ‘U-turn’ on their COVID-19 school exams replacement policies. After cancelling exams, they sought teacher estimates on their grades, but supported an algorithm to standardise the results. When the results produced a public outcry over unfair consequences, they initially defended their decision but reverted quickly to teacher assessment. We explain these developments by comparing two ‘windows of opportunity’ overseen by four separate governments, in which the definition of the problem, feasibility of each solution, and motive of policymakers to select one over the other lurched dramatically within a week of the exams results. These experiences highlight the confluence of events and choices and the timing and order of choice. A policy solution that had been rejected during the first window, and would have been criticised heavily if chosen first, became a lifeline during the second. As such, while it is important to understand *why the standardisation process went so wrong*, we focus on *why the policymaking process went so wrong*.

**Supplementary Information:**

The online version contains supplementary material available at 10.1057/s41293-021-00162-y.

## Introduction

In 2020, the UK and devolved governments performed a ‘U-turn’ on their COVID-19 school exams replacement policies. The experience was embarrassing for education ministers and damaging to students. The outcome was a policy that ministers rejected in March and criticised until August, then selected within days of the August exam results. This order of choice ensured that the eventual solution was in poorer shape in August because they had not invested in its technical feasibility from March.

All four governments exhibited remarkable similarities in relation to the confluence of events and order of key choices. This similarity began when they each responded to the COVID-19 pandemic by cancelling school exams and seeking an alternative way to assign grades to students. It continued when they identified two main solutions—(1) rely entirely on teacher assessed grades or (2) standardise the results—and selected the latter. As a result, they gave authority to exams agencies to design and implement an algorithm—i.e. a list of rules and step-by-step instructions to solve a problem—to produce standardisation (coupled with an appeals students for affected students). When the results produced highly visible examples of unfair consequences, they responded to intense criticism by reverting to teacher assessment in a matter of days. The outcome of a confluence of choices and events was a policy solution—teacher assessed grades—that was rejected during the first ‘window of opportunity’ for policy change but chosen quickly during the second.

We examine what happened in each case by asking: *what policy solution did each ‘window of opportunity’ produce,* and *how important was the order of choice?* The answer to these questions allow us to address a puzzling outcome: *why did each government reject and criticise then embrace the same policy solution so quickly?*

We design these questions with reference to three key concepts. First, multiple streams analysis (MSA) describes major policy change during a ‘window of opportunity’, in which there is high attention to a problem, a feasible solution is available, and policymakers have the motive and opportunity to accept it (Kingdon 1984; Herweg et al. 2018; Cairney 2018). In each nation, there was an *initial* ‘window’ for policy change following the pandemic and cancellation of exams, followed by a *second* window caused by the rise in public, media and policymaker attention to the problem of unfair exam results, which shifted the feasibility of two different policy solutions and gave politicians a new motivation to act.

Second, heresthetic describes the strategic context for decision-making, in which the *order of choice* and *time available to choose* can have a profound effect on outcomes (Riker 1986). A problematic policy solution was rejected during the first window then accepted wholeheartedly during the second. Although this solution would have been almost certainly been criticised heavily and widely if chosen during the first window, it became the only feasible solution and a lifeline to all four governments during the second. By then, from the perspective of policymakers under pressure to change policy quickly, there was no time to produce a well-designed policy for teacher assessment or consider new alternatives.

Third, studies of policy learning and transfer help describe the unequal UK and devolved government dynamic. They reinforce the sense that policymaking is not a rationalist exercise: learning is a political process and transfer relates strongly to the power of participants (Dunlop and Radaelli 2013, 2018; Dolowitz and Marsh 1996, 2000). On the one hand, UK and devolved governments made a commitment to a high degree of coordination and learning: a ‘four nations’ approach. On the other, we should not equate this process with gathering evidence of the impact of each other’s actions on the policy outcomes they claim to prioritise, such as to reduce inequalities of attainment.^1^ Rather, learning is a political process in which: the UK government tends not to follow the lead of devolved governments; and, while each devolved government has a different motive to follow the UK, the effect of UK government policy is impossible to ignore (Keating et al. 2012). Overall, the confluence of events and choices in each window, *plus* the order and timing of each window, *and* the varying influence of each government on each other, had a major impact on policy change.

Although each of the four UK nations provide distinct case studies of policy change, they produced very similar choices at similar times to respond to similar events. COVID-19 transmission and school closures provided the first impetus for a major change to exams policies, while the exam results crisis prompted the second change. The Scottish Government (overseeing an education system different from the others) was first to react because its exam results are published the earliest, while the subsequent UK decision had a disproportionate impact on Wales and Northern Ireland.

We treat each of their experiences as variations on the same theme to help narrate a process involving three MSA ‘streams’ times four nations times two windows. First, we explain the main elements of multiple streams analysis, heresthetic, and policy learning and transfer to show how the nature, order, and interaction between windows of opportunity matters. Second, we analyse government choices and statements—in the public record—by ministers and exams regulators (online Annex A^2^) to present a 4-nations account of two consecutive windows of opportunity, guided by the following questions:

1. During the first window prompted by COVID-19 and the cancellation of exams
  How did each government define the *initial* policy problem?
  Which policy solutions were initially feasible?
  What motivated each government to select one over the other?
2. During the second window prompted by policy failure and the exams crisis
  How did each government define the *next* policy problem?
  Which policy solutions were still feasible?
  What motivated each government to select a new solution?

Throughout, we reflect on a general sense of disorder that is difficult to exploit for political gain or correct in a straightforward manner. MSA and heresthetic studies often focus on policy entrepreneurs or exceptional actors, able to use their skills, networks, tenacity, and cunning to influence the process of choice. In this case, it would be outlandish to suggest that policymakers designed or entrepreneurs influenced the sequence of choice. Rather, many actors played a pivotal role during one stream or window, contributing unintentionally to the overall effect. As such, while it is important to understand *why the standardisation process went so wrong*, we encourage readers to focus on *why the policymaking process went so wrong*.

## Multiple streams: the window of opportunity for choices

We aim to explain a sense of policymaking disorder, involving two consecutive instances in which policymakers responded quickly to events not in their control and produced solutions that did not resolve the problem. MSA is a good initial fit because it provides a contrast to the classic idea of a ‘rational’ policy process turning well-defined and researched aims into outcomes via a series of orderly steps. In theory, there is a core group of powerful actors, and they define a problem, generate feasible solutions, and choose the best solution. In practice, the ‘garbage can model’ identifies ‘organized anarchy’ in which the order of steps is unclear and may be better understood as ‘relatively independent streams within an organization’ (Cohen et al. 1972, pp. 1–3).

Kingdon (1984) modified and applied this model to the US, showing that policymaker attention to a problem does not set in motion an inevitable process of policy change via orderly steps (Cairney 2020a, p. 196). Rather, major policy change is rare, and only occurs during a ‘window of opportunity’ in which three streams come together:

1. Problem stream: there is high attention to a particular way to define a policy problem.
2. Policy stream: there is already a technically and politically feasible solution to that problem.
3. Politics stream: policymakers have the motive and opportunity to select that solution.

This process *looks* straightforward, but only when described *in this order* and in relation to the *functional requirements* associated with ‘rational’ policymaking: we need policymakers to pay attention to a problem, produce feasible solutions, and select one. However, the gap between functional requirement and policymaking reality is wide in each case, and any one of the three streams can initiate a window of opportunity (Cairney 2020a, pp. 196–202).

First, Kingdon (1984, pp. 98–99) describes problem definition as an intense competition to harness scarce and fleeting policymaker attention. It is not an ‘evidence-based’ process, and attention does not relate to the ‘objective’ size of a problem (Cairney 2016, p. 42). It happens in a crowded political environment where the audience’s beliefs and interests, and events outside of anyone’s control, are more important than agenda setting strategies. Actors can seek attention for an issue in vain for long periods, only for attention to lurch for an indeterminate amount of time.

Second, since attention lurches quickly from issue to issue, it is too late to produce a viable solution after that shift. Attention to a problem can lurch in a day, but it can take years to produce a solution that is technically feasible (it will work as intended if implemented) *and* politically feasible (it will be acceptable to enough powerful people) (1984, pp. 138–146). Kingdon (1984, p. 18) describes a gradual process in which some actors propose ideas while others modify them before they become acceptable in policy networks. This disconnect between lurching attention and slow policy development produces a dynamic of ‘solutions chasing problems’. Actors develop solutions in anticipation of future problems, then find the right time to encourage and exploit attention to a problem.

Third, policymaker beliefs limit their attention to problems and their receptiveness to solutions, and their opportunities to act are limited. Kingdon (1984, p. 19) describes factors key to their motive and opportunity to act—including ‘vagaries of public opinion, election results, changes of administration … and interest group pressure campaigns’—but each can encourage or discourage policy change.

Crucially, the impetus for policy change can come from any of the three ‘streams’: a new government has new ideas, a crisis prompts attention to a problem, or promoters of a solution generate new support. Further, there is *some* potential for powerful actors to encourage such developments. Kingdon (1984, p. 173) describes ‘policy entrepreneurs’ in that context. Entrepreneurs possess knowledge, connections, and tenacity, but also rely on luck to secure their policy solutions: ‘advocates lie in wait in and around government with their solutions at hand, waiting for problems to float by to which they can attach their solutions, waiting for a development in the political stream they can use to their advantage’ (1984, 165–6). In other words, the policymaking environment constrains and facilitates action, and actors are relatively successful when they have the resources to adapt and respond well (Cairney 2018).

MSA is one of the most applied policy theories (Heikkila and Cairney 2018). Most use MSA metaphorically to highlight the serendipitous events and choices that contribute to infrequent major policy changes (Jones et al. 2016; Cairney and Jones 2016). The garbage can image highlights messy processes in which policymakers may not fully understand a problem, know how to solve it, or have enough time to process policy-relevant evidence and gain clarity: ‘Yet, choices are made, problems are defined and solutions are implemented’ (Zahariadis 2003, p. 1). More precise studies highlight the need for many conditions to be met at the same time to enable policy change (Shephard et al. 2020; Herweg et al. 2018). Further, the growth of MSA studies beyond the US highlights variations in experience, in which solutions may become feasible more quickly if developed successfully by other governments (particularly when action by one government puts pressure on another to act), and entrepreneurs can have more influence if operating in smaller environments (Cairney and Jones 2016). Still, there remains an emphasis on policymaking environments out of the full understanding its participants. Actors can exploit events but contribute to outcomes over which no-one has full control.

## Heresthetic: the order of (and time to make) choices

MSA can only take us so far. Kingdon (1984) did not anticipate multiple consecutive windows of opportunity or windows in multiple jurisdiction. Few MSA studies examine them, and none examine two such closely related windows involving four different governments.

The closest example is Zahariadis’ (2003, pp. 32–4, 84) study of a succession of windows of opportunity to privatize UK industries before policy change finally happened. Such accounts highlight necessary but insufficient conditions, in which attention to a problem may rise, but a feasible solution does not yet exist, or policymakers do not have the motive to act. It warns us against treating a major policy change as quick or inevitable, particularly if we can identify many previous instances of missed opportunities. It can take decades to see the spark of an idea translated into policymaker action. In contrast, our case studies are characterised by: two consecutive instances in which governments *did* act during each window *in the same year*; and, the first action became responsible for the dynamics of choice during the second window (policy became ‘its own cause’, Wildavsky 1980, p. 62).

Therefore, we need a way to analyse the profound impact of one window on the limits to choice in another. Although it would be silly to suggest that someone planned these events, it makes sense to analyse them through that lens. The order and timing of policymaker choice matters even if no single person is responsible for its manipulation.

Riker (1986, p. ix) uses the term ‘heresthetic’ to describe this manipulation: ‘structuring the world so you can win’. People ‘win politically because they have set up the situation’. We may not be able to change people’s beliefs, but we can ‘exploit the fact that they possess many different—and often contradictory—preferences’ (Cairney 2020a, p. 65). For example, Riker (1986; see also Cairney 2021; McLean 2002; Drew 2019; Joseph and Fahey 2018) shows that it pays to:

1. Design the order and way people make choices.
  Many policy preferences are ‘intransitive’: if A is preferred to B and B to C, A is not necessarily preferred to C. Even if people’s fundamental preferences do not change, a choice that would be rejected in one context may be welcomed in another.
  Strategies include:
  Design decision-making rules to consolidate support for your option while splitting support for others.
  Compare a small number of options to make sure that yours is the most competitive.
  Design a series of votes, in sequence, to allow you to trade votes with others.
2. Exploit how people deal with their own cognitive limits.

No-one can process all information. Instead, they combine cognition and emotion to make efficient choices in high pressure political environments (Cairney and Kwiatkowski 2017).

Strategies include:

- Design the framing of choice and selection of criteria to evaluate options (for example, is it about protecting the credibility of the system, trusting professionals, or fairness to students?)
- Only draw attention to some problems and solutions.
- Make your preferred problem framing or solution easy to understand.
- Make other solutions difficult to process, such as by presenting them in the abstract and providing excessive detail.
- Emphasize the high cost to examining other options.
- Ensure that people make choices quickly, to ward off the possibility of further analysis and risk of losing control of the design of choice.

No actor manipulated choice in this way, but the order and limits to choice had a major impact on exams policy. Policy crisis and failure combined to produce limits to policymaker attention, attention to a small number of options, and a sequence of events that shifted the relative feasibility of each choice.

## Policy learning and transfer: the dynamics of coordinating choices

We compare four separate ‘windows’ because four governments are responsible for education policy across the UK: the UK government for England, and the devolved governments in Northern Ireland, Scotland, and Wales. Yet, we also consider how they learned from each other and made similar policy choices.

UK and devolved government narratives emphasise *orderly*, *routine*, and *rational* learning: all four made a commitment to learn from (or at least consult) each other as part of a ‘four nations’ approach during the early stage of the COVID-19 pandemic (Cairney 2020b). In that context, the UK government performs a coordinative role to reflect its wider powers (such as to provide economic support for policy) and greater capacity to share policy-relevant COVID-19 evidence. Welsh and Northern Ireland governments also highlighted the importance of coordinating decisions to ensure that their students were not disadvantaged by the timing of choices in other jurisdictions (Birt 2020). The exams agencies also describe parallel discussions on contingency planning between Ofqual and ‘representatives from each of the exam boards, the JCQ, the Department for Education, UCAS and qualifications regulators from Northern Ireland, Scotland and Wales’ (Ofqual 2020a, p. 27).

In contrast, policy studies suggest that policy learning and transfer are *political* processes. Forms of policy learning include ‘epistemic’ (policymakers learning from experts) and ‘reflexive’ (via wider participation and deliberation), but also ‘bargaining’ (political actors learning how to win) and ‘hierarchy’ (to learn how one government can influence others) (Dunlop and Radaelli 2013, 2018). Further, the impetus for policy transfer can range from relatively voluntary (the choice to study and follow other governments) to relatively coercive (the obligation to emulate the choice of other governments) (Dolowitz and Marsh 1996, 2000). Within this range is ‘indirect coercive transfer’ (1996, p. 348), when one government feels obliged to respond to policy by another.

The latter is a strong feature of UK and devolved government relations. The UK government rarely feels pressure to follow the lead of the others (it looks to experiences of larger countries), but each devolved government feels some pressure to relate policy to the UK (see Keating et al. 2012 for case studies). As such, a ‘four nations’ approach is pursued by governments with unequal powers, with a tendency for UK government choices to spill over to—and limit the options of—the others (and Wales and Northern Ireland in particular).

## The first window of opportunity: dealing with COVID-19

Initial choices demonstrate high cooperation. The decisions to close schools were ‘tightly coordinated’ and announced on 18th March 2020 (Sargeant 2020, p. 4; Weale 2020; BBC News 2020a; EANI 2020; Adams and Stewart 2020). Global comparisons suggest that these school closures and exams cancellations were not inevitable, but closures were very common in countries with very high rates of COVID-19 infection and transmission (International Association for Educational Assessment 2020). These choices were made by actors external to education policy communities, focusing primarily on public health. Therefore, we could describe *three* sequential windows of opportunity: to close schools and *delay* or *cancel* exams, select a replacement, then replace the replacement. However, we treat school closure primarily as an event—not in the control of education departments—that prompted the first search for a substantive policy solution.

### How did each government define the initial problem?

The closure of schools created a major problem for examinations policies, defined by each government as: how to apportion grades to students who were unable to sit crucial examinations, and set up a system remarkably quickly. Each government dismissed quickly the idea of *delaying* exams, on the assumption that schools could be closed for 3–6 months and this amount of delay would disrupt the transition to tertiary education.

In this regard, there are differences between (and often within) the four nations in terms of the structure, timing, weight, and relationship between the different examinations (Wyness et al. 2013; Sibieta 2019; Adams 2020b), and Scotland’s exam structure is particularly distinctive. However, in general, their A-level (England, Northern Ireland, Wales) and Higher/ Advanced Higher (Scotland) examinations have similar policy implications: they dictate entry to further and higher education (and influence employment opportunities). Students engage in continuous assessment to some extent, but in-person examinations form a large weighting for the calculation of final grades.

Each government defined the policy problem in relation to the need to replicate the purpose of exams to allow students to progress to tertiary education or employment. All four announced their intentions to *allocate* grades to students:

- Scottish Education Secretary John Swinney announced (18^th^ March) that the Scottish Qualifications Authority (SQA) had started to develop an alternative certification model (BBC News 2020b).
- UK Education Secretary Gavin Williamson stated (18th March): ‘we will not go ahead with assessments or exams, and we will not be publishing performance tables for this academic year. We will work with [the] sector and have to ensure children get the qualifications that they need’ (Adams and Stewart 2020).
- Welsh Education Minister Kirsty Williams announced (18th March) that students would, in lieu of examinations, receive a "fair grade to recognise their work, drawing on the range of information that is available" (quoted in Evans 2020).
- Northern Ireland Education Secretary Peter Weir (19th March), gave 'an absolute assurance that the qualification will be there … Very good work has gone on by CCEA [Council for the Curriculum, Examinations & Assessment].. we are reaching a situation where all of the examining boards will be in-step' (Murray 2020).

Each government expressed a preference for retaining the long-term ‘credibility’ of the examinations system:

- Scottish First Minister Nicola Sturgeon highlighted the importance of the system producing a ‘credible—and that’s important for young people—system of results’ (quoted in Lee 2020).
- Williamson pointed to the importance of ‘mitigating the risk to standards’ of relying solely on CAGs (‘Centre Assessment Grades’ in E&W; ‘assessed in S&NI)) (Williamson 2020).
- Williams described ‘a fair and robust approach to issuing grades to the summer 2020 cohort’ (Williams 2020).
- Weir highlighted the importance of ‘robustness’ in allocating grades (McHugh 2020).

In all four systems, this definition in relation to ‘credibility’ and ‘robustness’, with less emphasis on trusting teachers or clarifying the meaning of ‘fair’ results, related to the international image of its school system and its *connection to tertiary education*. Although their secondary education systems are separate, the tertiary system is less so. Each government had an incentive to keep the examinations that dictated university entry broadly in line with previous years, to avoid unintended consequences for UK universities which accept large numbers of students from across the UK. There was some incentive for the four governments to operate similar systems and keep grades broadly in line with previous years, to ensure a level of predictability in student recruitment (Adams 2020a). Indeed, the university connection had reduced the political feasibility of delayed school exams.

### Which solutions were initially feasible?

This problem definition was key to the feasibility of each potential solution. The four governments considered two main possibilities relevant to a ‘credible’ response. First, *rely solely on teacher grading* (Centre Assessed Grades, CAGs). CAGs are ‘based on a range of evidence including mock exams, non-exam assessment, homework assignments and any other record of student performance over the course of study’ (Harrison 2020). Issues with technical (and political) feasibility include:

- The potential for high variation and discrepancies between (a) different centres and (b) marking within centres, in which different teachers weight their decisions differently or enjoy different resources to do so with accuracy. Wyness and Murphy’s (2020) analysis of predicted grades finds that ‘only 16% of [English] students received accurate predictions for all three [A-level grades], with 75% overpredicted and just 8% underpredicted’.
- The potential for higher predicted grades leading to an unusually high number of students achieving sufficient grades to enter university, producing the potential for overload of the sector. This effect would be felt unequally across English universities, since UK Education Secretary Williamson had announced that they would be subject to a cap on domestic student entrance numbers based on their forecasts for the next academic year plus 5% (Department for Education 2020; UK Government 2020a).
- Predictions benefit higher performing students, who tend to be from more privileged backgrounds. Wyness (2016) describes evidence of ‘socio-economic (SES) gaps in predicted grades at A-Level in England: among the highest achieving students, those from disadvantaged backgrounds receive predicted grades that are slightly lower than those from more advantaged backgrounds’. Evidence collected by the House of Commons Education Committee showed that there were serious risks that ethnic minorities, disabled people, and low income students were at risk of systemic bias from processes drawing on teacher prediction (House of Commons Education Committee 2020). Further, a Department for Education evidence review suggested that predicted grades often lead to (albeit small) differences between teacher assessment and exam assessment results in relation to ‘gender, special educational needs, ethnicity and age' (Lee and Walter 2013).

Second, *take CAGs as a starting point then use an algorithm to aid ‘standardisation’*. Each government considered *the technical feasibility* of this option in a different context (and the availability of this data differed between qualification level and national jurisdiction). In Wales and Northern Ireland, AS Levels (in year 1) formed part of the basis of A-Level results (years 1 and 2), which allowed their qualifications agencies to use this information to produce a more ‘accurate’ simulation of student grades (Welsh Government 2020b; nidirect 2019). In England and Scotland, there is no such relationship between the two—formally separate—qualifications, meaning a ‘standardised’ grade would have to rest on other data, such as student attainment in coursework towards the qualifications and mock examinations at the individual student level (Annex A: Table 2).

However, all governments performed similar (and ultimately incorrect) assessments of the *political feasibility* of this option (not anticipated by governments), in which regulators also drew on data pertaining to the gap between *school* predicted and actual grades to represent the students’ ability in a wider context. This way to calculate grades was technically and politically feasible *at the time*. Each government was attracted to the general idea of standardisation, which allowed students to complete their secondary education and to progress to the next level in similar ways to previous (and future) cohorts. Further, an emphasis on the technical nature of this standardisation, fostered by delegation to examinations agencies and a decision to not share the details of its algorithm was a key part of its temporary political feasibility.

### What motivated each government to select one over the other?

All four governments were driven by the choice to close schools, cancel exams, and find a way to reproduce the results so important to the credibility of the education system (primarily in relation to avoiding ‘grade inflation’) and entry to tertiary education. They each relied heavily on agencies tasked with designing the new system and a relatively small community of assessment experts (Annex A: 1–3). They chose to announce a process of standardisation to the CAGs provided by teachers, often backed by expert commentators and the agencies responsible for administration. For example, advocates of standardisation included Dennis Sherwood, a qualifications commentator and former Ofqual employee, arguing that standardisation done ‘well by teachers with integrity is the fairest method, and beats the reliability of the current exam system hands down’ (quoted in Lightfoot 2020). The National Education Union stated that ‘it helps with consistency and fairness to have moderation and oversight from the regulator’ but signalled discomfort with the potential for ‘rationing of success’ represented by the system, and the requirement for students to be ranked by teachers (NEU 2020). Paul Whiteman, the General Secretary of the Association of Headteachers also offered qualified praise for Ofqual's proposals, stating on April 3rd that *students were in the 'safe hands' of professionals* (Adams 2020b).

In Scotland, Education Secretary Swinney restated the infeasibility of holding exams (even if they could be delayed) and told the Scottish Parliament (19th March) that he ‘anticipate[s] that this model will use coursework, teacher assessment of estimated grades, and prior attainment as the basis for certification’ (Peterkin 2020). The Northern Irish and Welsh Education Secretaries made similar announcements the following day (McHugh 2020; Evans 2020), while the UK Government followed shortly afterwards, announcing that a ‘fair grade’ would be produced when ‘teachers will take into account a range of evidence and data including performance on mock exams and non-exam assessment’ (UK Government 2020b).

In other words, all four governments made similar claims when defining the problem and selecting each solution. They describe *their own responsibility for pursuing the high political feasibility of standardizing test results* to address teacher grade inflation and maintain the exam system’s credibility, and the *delegation of technical feasibility*—to design the algorithm to that end—t*o expert agencies*. They each identify their political authority to pursue standardization then delegate technical authority to exams agencies, providing a general assurance that the algorithms would work as intended without revealing the details in advance.

As such, the cohesion of these actions and messages is notable in two respects. First, ministers were motivated by different policy aims that made standardisation a more-or-less attractive aim, and this ideological variation adds significance to the similarity of solutions:

- Seeking to avoid grade inflation appealed particularly to the centre-right Conservative party which had made this objective a centrepiece of recent English educational reform. Former Education Secretary Michael Gove oversaw reforms to mitigate against grade inflation, arguing in 2013 that improved grades ‘flattered’ Ministers but didn’t benefit children (Gove 2013).
- It was a harder sell to the SNP which had positioned itself as a social democratic political party committed to education equity. First Minister Nicola Sturgeon had placed education policy at the forefront of its governing agenda, asking for her government to be judged on its record in closing the attainment gap between the wealthiest and most deprived Scottish school students. However, its algorithm seemed to rule this aim out by tying a student’s results to the historic results of each school. The same student grade could be reduced more in a school with historically lower grades, and lower grades are more likely to be found in schools in more deprived areas (McNab 2015).
- Wales is run by a Labour-led centre-left coalition (with a Liberal Democrat education minister). *In opposition*, the (UK) Labour Party was critical of standardisation in England and Scotland (Adams et al. 2020; Hepburn 2020), but *in government* Welsh Labour defended the move.
- Northern Ireland is governed by a coalition of the main parties in a ‘power sharing’ agreement led by the centre-right Democratic Unionist Party and centre-left Sinn Fein.

Second, the specifics of the algorithm were not announced immediately. This approach reduced (a) debate on its unequal impact on students and (b) the chance for other experts to examine if the algorithm’s rules and instructions would produce the desired effect. Subsequent announcements with more detail (Annex A: Table 2) generated mild opposition and technical debates by experts. Pre-publication discussion was largely hypothetical, generating modest concern for the unequal impact of standardisation on different individuals and groups (Ferguson 2020), and with early inquiries more concerned with the inequity of teacher assessment (House of Commons Education Committee 2020). Policymakers in all four governments (aided by fairly sympathetic media coverage) assured students that the grading would be accurate and fair, with teacher discretion playing a large role in the calculation of student grades (ITV News 2020).

### The outcome of the first window of opportunity: ‘standardised’ results

COVID-19 prompted the UK and devolved governments to close schools for over six months, opening a window of opportunity to find a new school examinations policy. All four governments:

- Defined the problem as the need to produce a credible outcome.
- Considered teacher assessed grades and standardisation.
- Were motivated to select standardisation to foster credibility in relation to previous exam results and smooth progression to tertiary education, and to reflect their view (based on expert feedback) that standardisation would iron out the inconsistencies, inaccuracies, and inequalities associated with teacher discretion.

In other words, all four governments aligned at versions of the same policy, at roughly the same time, for roughly the same reasons, to follow the same basic motivation. There are important variations on this theme (Annex A: Table 1), but the ability of each government to go its own way accentuates the levels of policy similarity that still occurred. There was also a process of policy convergence, with a high incentive to coordinate policy as part of a general ‘four nations’ approach and specific desire to manage the student transition to tertiary education. At that time, the ‘national mood’ was not hostile to the standardisation process. To these governments, it *appeared at first* that they had found a fair and efficient (or at least defendable) way to allocate grades in the context of enforced, indefinite school closure at a time in the school year where wiggle room was limited.

## The second window of opportunity: dealing with the impact of the first choice

However, these appearances proved to be deceptive, and they vanished on each day of each exam result. A rapid shift in the ‘national mood’ (Kingdon’s shorthand, used here to describe intense public, media *and* parliamentary attention) opened a new window of opportunity within five months of the first (beginning first in Scotland, since its exam results on 4th August preceded those of the others on 13th August). We use the three ‘streams’ as subheadings to narrate these events even though they overlap in practice, not least because the shift in national mood (politics stream) motivated each government to redefine its policy problem. This way allows us to include in the ‘problem stream’ a discussion of problem definition as a collective or competitive process to set the agenda. In the ‘politics stream’ we explore the dual motivation of policymakers: (1) to address this policy problem and (2) solve their own political problems, including external pressure on education ministers to resign.

### How did each government define the next policy problem?

Each government defined its new problem in relation to policy failure and the need to solve the unintended consequences of its previous policy. It began with the release of exam grades in Scotland. The Scottish national mood shifted so intensely that, after a few days, pursuing standardisation no longer seemed politically feasible. There was some general concern about the scale of standardisation, since the outcome ‘resulted in 124,564 of the 511,070 grades awarded being marked down [..] in many cases this proved to be the difference between a student passing or failing their subject, with 45,454 grades being cut from an A-C pass to a D or below’ (Green 2020). However, the main effect of intense public, media, and parliamentary attention was to define the policy problem specifically as one of unfairness to students in relatively deprived areas (measured in relation to the Scottish Index of Multiple Deprivation). Crucially, this criticism related primarily to:

1. the unequal level of *reductions of grades after standardisation*, caused largely by connecting current and previous performances in the same school (disadvantaging poorer students), rather than
2. the unequal *overall rise in grade performance* after teacher assessment *and* standardisation (advantaging poorer students).

For example, following SQA standardisation, the pass rate awarded by teachers to the students in the Most Deprived areas fell by 10.5% in National 5s (equivalent to GCSEs), 15.2% at Higher and 10.8% at Advanced Higher (equivalent to A levels). Meanwhile, students from the Least Deprived areas saw their pass rates reduced by far less: 5.2%, 6.9% and 7.3%, respectively (SQA 2020). This way to breakdown the results and emphasise the iniquitous socioeconomic distribution was common in social media debate but did not win the day in government *immediately*, partly because it emphasised only one part of the process. For example, the *overall* effect on A-C grades at Higher was a **7% overall improvement in the Most Deprived areas** (+ 4.6 percentage points, from 65.3% in 2019 to 69.9% in 2020) even though standardisation reduced this score from an estimated 85.1%, while there was a **3–4% overall improvement in the Least Deprived areas** (+ 2.9 percentage points, from 81.7% to 84.6%, which was downgraded from an estimated 91.5%) (Table A13, SQA 2020). Further, Sturgeon argued that the adjustments had been ‘necessary to make sure we have a credible—and that’s important for young people—system of results’ (Lee 2020).

Nonetheless, the backlash was felt immediately, with the Scottish Government coming under sustained criticism from unions, students, parents, and opposition parties in the Scottish Parliament. Scottish Labour accused the Scottish Government of ‘marking the school, not the pupil, and baking in the attainment gap’ (quoted in Stewart 2020). The author Amna Saleem (tweet by @AGlasgowGirl [https://twitter.com/AGlasgowGirl/status/1290570050542460928] quoted in Carrell 2020) provided an exemplar of experiences of class based discrimination, describing ‘an absolutely defeated baby brother over here who did well in his prelims only to be completely shafted by the SQA who seem to be rounding down at least two bands across the board in what looks like a dedicated attempt to throw working-class kids under the bus’. On August 7th, around one hundred students gathered in Glasgow's George Square, holding signs protesting the ‘postcode lottery’ of the downgrades and urging the Scottish Government and SQA to revert to the (higher) teacher predicted grades. TV and print media highlighted stories of students in deprived areas achieving consistent **A** performances but being marked down heavily after standardisation, such as Erin Bleakely (the organiser of the protest) who saw her maths grade drop from an **A** in her prelimin to a **D** in the final results (BBC News 2020c).

This shift of problem definition did not happen in the rest of the UK until their own published exam results highlighted similar problems. As in Scotland, downgrades were concentrated in more deprived areas, prompting government ministers to emphasise the overall result (largely in vain) while external attention lurched more specifically to the many students (with expected high grades) who were downgraded according to circumstances outside of their control.

In England, 36% of grades were downgraded, with disadvantaged pupils shown by Ofqual official statistics to be worst hit, while private schools increased the proportion of students achieving A and A* grades. The overall effect on A-C grades for ‘low socio-economic status’ students was a rise from 51.8% in 2019 to 54.7% even following Ofqual's moderation (Ofqual 2020b, p. 150). However, the year-on-year rise in proportion of students achieving A or A* grades was much higher at independent schools than state comprehensives (Adams and McIntyre 2020). It led to sustained criticism from media commentators, political opponents, and protests by students (Sabbagh 2020; Bates 2020). Students were portrayed sympathetically, particularly when the fate of high achieving students from deprived areas clashed with the UK Government’s rhetoric to ‘level up’ across the country. On August 15th, Williamson defended a ‘fair and robust system for the overwhelming majority of students' (Swinford 2020).

In Wales, 42% of students saw their A-Level results lowered from their CAGs (Express and Star 2020), while First Minister Mark Drakeford highlighted the success of students, pointing to an increase in the number of high grades, and Education Minister Kirsty Williams described a ‘very fair and robust’ system of apportionment (BBC News 2020d). In Northern Ireland, a third of students saw their A-Level grades downgraded, but ministers followed the same path as the other UK nations, initially defending the apportionment and drawing attention to the overall distribution of grades, which was slightly stronger results than previous years (BBC News 2020e).

### Which policy solutions were still feasible?

Each government faced similar choices between a very small number of suboptimal solutions:

1. Defend the original system, finding ways to challenge the newly dominant narrative on unfairness, and emphasising the ability of students to appeal unfair results.
2. Modify the original system by changing the criteria of the appeal system, to boost the number of allowable appeals and likelihood of success.
3. Abandon the original system and revert entirely to teacher assessed grades.

The technical *and* political feasibility of each option was unclear. A reliance on appeals seemed possible, but each qualification agency would be dealing with a far larger than expected number, contributing to major delays, processed under the public spotlight, and with major knock-on effects for university admissions (universities confirm their offers to students almost immediately after the results are released). A shift to teacher assessed grades would produce a major boost in exam results, contributing to ‘grade inflation’ (by that time, everyone knew that the results would be unusually high), and have a different knock-on effect for university recruitment (for example, prompting universities to seek the removal of a financial cap on recruitment in a single year). Further, an increase in A-Level (and Higher) grades would lead to a great proportion of students achieving entry to their first choice university, with smaller universities losing out proportionally. Crucially, it would represent the reversal of *an argument against teacher assessed grades that was actually stronger in August than in March* (since no government was able to invest resources and planning to get this solution right in advance).

### What motivated each government to select a new solution?

Each government was motivated by the need to respond quickly and effectively to a major public, media, and parliamentary backlash that threatened the credibility of the system, trust in the competence in government, and the careers of education ministers.

In Scotland, Swinney announced the ‘U-Turn' on 11th August. The embrace of teacher assessed grades quickly became the only politically attractive option for a minister under pressure to resign and a government seeking to protect its centre-left credentials in relation to a (1) wider public that, until August, had expressed high faith in the competence of the Scottish Government to address COVID-19 (McCall 2020), and (2) predominantly left-wing education policy community, membered by trade unions and education professionals (Paterson 2017, p. 146; Cairney 2013), and active during the immediate outcry. For instance, the main teaching union EIS’ General Secretary Larry Flanegan had cautioned the Scottish Government against modifying teacher allocated results and welcomed the change of course with reference to his own organisation’s prescience (EIS 2020).

The other parts of the UK were watching closely, but they maintained their approach until a similar public backlash appeared in their own jurisdictions. In the meantime, one concession by the UK Government (12th August) was a ‘triple lock’, whereby ‘students could accept their calculated grade, appeal to receive a valid mock result, or sit autumn exams to ensure the achievements of young people are recognised’ (UK Government 2020c). The timing (on the eve of the release of A-Level grades) is telling, suggesting that UK ministers were mindful of the potential for a repeat of Scottish events in England. Yet, this intervention was not enough to stave off a similar reaction in England, with Results Day acting as a second crisis for policymakers.

Ultimately, all three governments quickly followed the same path. Initially, they opted to defend their original policy choice. However, by 17th August, the UK, Welsh, and Northern education secretaries announced (separately) that examination grades would be based solely on CAGs unless the standardisation process had generated a higher grade. Although ostensibly separate decisions, the Scottish Government context and—in particular—UK Government ‘U-Turn’ increased the likelihood that the Northern Irish and Welsh governments would follow suit (and coordinate the communication of their responses). Weir described this process during the Northern Ireland Assembly ‘AS-level and A-level Grading Crisis’ debate (Official Report 18.8.20: 2.15 pm):

There were discussions between ourselves and England. Wales was seeking a similar approach. It was not simply a question of us following England, but whenever we have a situation where the English market represents about 85% of students in the UK as a whole, we simply could not go in some solo direction. So, yesterday, we ended up announcing at exactly the same time as England. Wales announced roughly about an hour before us, but all three nations were kept in step. Whatever other concerns there are, we now have a situation where all parts of the United Kingdom are in exactly the same position as regards all the qualifications.

Further, Welsh Government policymakers made clear that their motive was less the failure of their own system, which they continued to defend, but the domino effect of other governments reverting to CAGs (Birt 2020).

### The window of opportunity for a policy ‘U-turn’ and domino effect

A confluence of factors persuaded policymakers to change course. The most notable was a rapid shift in the ‘national mood’ triggered by the publication of grades and hostile press coverage given addition weight by the testimonies of students. The main focus was an impersonal process with unfair results. As the Scottish Government's review stated, “The algorithm does not [care] that the data are individuals; it would move the data around until the ‘optimal’ distribution was achieved” (Priestley et al. 2020, p. 43). The dominant image involved sympathy for the victims of an impersonal process which took no account of their individual talents. Often, they were deemed as gifted and hardworking, suffering the misfortune to attend a lower-performing school, being punished despite overcoming poor odds, and losing out on offers of admission from ‘desirable’ universities (Oxford, Cambridge, or ‘Russell Group’). The inability to identify these ‘outliers’ automatically undermined the technical feasibility of the system and made it increasingly difficult to defend, exacerbating the more general problem in which inequalities in attainment are built into the algorithm that connects predicted results to school performance.

Somewhat ironically, some policymakers were able to reframe the problem by (1) identifying the consequences of their own (and their agency’s) failures, to (2) justify a change of course in Scotland and England:

- Swinney acknowledged that ‘the results left many young people feeling that their future had been determined by statistical modelling rather than their own capability and capacity’ (Scottish Parliament Official Record 11th August 2020). Sturgeon described the reversal by acknowledging that the standardisation process ‘meant that too many have lost out on grades that they think they should have had and also that that has happened as a result not of anything they’ve done but because of a statistical model or an algorithm, and in addition that burden has not fallen equally across our society’ (Merson 2020).
- Williamson stated that it ‘became increasingly apparent that there were too many young people that quite simply hadn't got the grade they truly deserved’ (quoted in BBC News 2020f).

Scotland’s initial experience was instructive to the rest of the UK; its example provided the UK government with a blueprint to follow (eventually). It began with a new policy choice—reverting to teacher assessed grades—sold as fairer to victims of the standardisation process. Once this precedent was set, it became difficult to resist, particularly when faced with a similar backlash. The UK’s government’s decision then influenced the Welsh and Northern Irish governments. In particular, Drakeford acknowledged this interdependence when stating that ‘once we knew changes were happening elsewhere, we were obliged to make these changes in Wales’ (Birt 2020), and pointing to the potentially unfair impact on Welsh university applicants. The latter represents a form of policy transfer which, while voluntary, relates strongly to the spill-over effects of a UK government decision (Keating et al. 2012; Dolowitz and Marsh 2000).

## Conclusion

The four governments of the UK produced two major changes to education exams policies in 2020. The first was to introduce standardised teacher assessment grades following the closure of schools and need to find an alternative to exams. The second was to remove standardisation within one week of the publication of the exam results. We describe this shift as a ‘U-turn’ to reflect widespread commentary on this episode, and to signal a major policy change. We describe a ‘fiasco’ because the eventual outcome is a policy that each government criticised, rejected, and largely neglected, before they welcomed it with open arms. Had they selected this policy in the beginning, they could have invested more resources in its planning, delivery, and consistency. Rather, in choosing it at the end, they embraced its unintended and often unknown consequences (such as on inequalities of attainment).

In that context, why did each government reject this policy solution so strongly then embrace it so wholeheartedly? The answer can be found in the order and timing of events and choices during two consecutive windows of opportunity.

In the first window, each government cancelled examinations and defined the next policy problem primarily in relation to the credibility of the exams system, to avoid grade inflation and its knock-on effect for its reputation and transition to tertiary education. This definition boosted the feasibility of a standardised system and undermined the feasibility of high teacher discretion to estimate a student’s likely grades. Policymakers in each government were motivated to act quickly and decisively to follow expert professional advice (in assessment and accountability, not teaching). At this time, they used general assurances about the technical feasibility of standardisation—via an algorithm whose rules were not known widely—to boost the political feasibility of their choice.

In the second window, each government responded to policy failure and a major public, media, and parliamentary outcry. They defined the policy problem primarily in relation to the unintended consequences of standardisation, in which the system was unable to detect students whose achievements would be downgraded by an algorithm that tied current results to the previous results in each school (and ‘baked in’ socio-economic inequalities). In other words, issues of technical and political infeasibility became mutually reinforcing. This new definition altered the feasibility of the next option, with a modified version of current policy becoming increasingly unfeasible as new problems emerged with its administration. Policymakers were motivated to find a politically feasible solution that ‘solved’ the policy problem *and* maintained the credibility of each government. As such, the solution that they rejected at first became a lifeline by the end. By August, it was a *technically worse* but *politically better* option.

This focus on order and timing should not be lost during the inevitable inquiries and reports on the UK and devolved examinations systems. The take-home message is to not ignore the policy process when evaluating the long-term effect of these policies. The initial instinct of governments was to commission research to evaluate *why the standardisation process and algorithm went so wrong*, and this evaluation will provide the chance to explore the use of non-transparent algorithms to deliver policy choices. However, we should also focus on *why the policymaking process went so wrong*, to produce a wildly inconsistent approach to the same policy choice in a short space of time. Examining both aspects of this fiasco will be crucial to the grading process in 2021, given that the UK and devolved governments will be seeking an alternative to exams for a second year (Annex A: 12–13).

## Supplementary Information

Below is the link to the electronic supplementary material.

Supplementary Information 1 (DOCX 44 KB)

## Footnotes

1 This article forms part of a wider focus on ‘spatial justice’ and ‘territorial inequalities’ in the EU (including the impact of public services on inequalities), funded by Horizon 2020/European Research Council [Grant Number REVINEQUAL-07-2016].

2 Annex A can also be found at https://paulcairney.files.wordpress.com/2021/01/annex-a-covid-19-exams-fiasco-across-the-uk-revised-15.1.20.pdf.
