# Supplementary material for: The COVID-19 exams fiasco across the UK: four nations and two windows of opportunity
Source: Br Politics. 2021 Feb 19;17(1):1–23. doi: 10.1057/s41293-021-00162-y (PMC7894241; doi:10.1057/s41293-021-00162-y)
Supplement: Supplementary file 1 — Supplementary Information 1 (DOCX 44 KB) [file 41293_2021_162_MOESM1_ESM.docx]

# **Annex A The COVID-19 exams fiasco across the UK**

## **Table 1: Exam regulator decisions and announcements 2020**

|  | **Describing the system** | **Defending the system** | **Explaining its demise** |
| --- | --- | --- | --- |
| **Scotland** | [On 20th April](https://www.sqa.org.uk/sqa/93920.html), SQA announced their system to enact Swinney’s request for a new examinations system to operate in lieu of in-person assessments. It would rest partly on teacher assessment. A moderation system would assess a school’s former performance (which a prior consultation process with teachers had identified as a potential problem). The SQA highlighted the importance of a new appeals process to address unintended consequences. | Before the Scottish Parliament Education and Skills Committee on the [12th August,](https://www.parliament.scot/parliamentarybusiness/report.aspx?r=12739&i=115206) SQA Chief Executive Fiona Robertson argued that moderation – partly by using historic schools data - was required because more than 50% of teacher predictions had historically been incorrect. Robertson argued that the SQA had been asked to devise a new system and had done so faithfully (while acknowledging the ‘anomalous’ impact on some students) | Following the Ministerial direction of the 11th August which reversed course, [the SQA announced](https://www.sqa.org.uk/sqa/94798.html) the following day that they would issue new grades based on teacher assessment, or the moderated grades in the event that these individual grades were higher. The SQA noted that they would publish the new grades quickly. The results were confirmed on the [31st August](https://www.sqa.org.uk/sqa/94978.html), with an SQA statement drawing attention to the ministerial directions which had dictated and framed their actions. |
| **England** | [On May 22nd](https://www.gov.uk/government/news/ofqual-gcse-and-a-level-consultation-outcomes-and-autumn-exam-series-proposals), Ofqual announced that students would be apportioned grades in lieu of examinations. The government consultation seemingly endorsed the proposed system, which rested on: teacher submission of grades, a ranking of students within cohorts, and a standardisation process. Ofqual also announced a planned additional exam series in the autumn for students unable to receive a calculated grade. | [On August 14th](https://dfemedia.blog.gov.uk/2020/08/14/misleading-a-level-claims-debunked/) (1 day after A-Level results), Ofqual chsallenged “misleading claims” in the media. It claimed that, contrary to the media narrative: 40% of pupils didn't have grades downgraded; the Government trusts teachers; the standardisation was introduced following consultation; and pupils from disadvantaged backgrounds weren't disproportionately affected. | [On August 17th](https://www.gov.uk/government/news/statement-from-roger-taylor-chair-ofqual), the Chair of Ofqual Roger Taylor announced that students would receive their Centre Assessed Grades (or standardised grade - whichever was higher). This would apply to A Levels, AS Levels, and GCSEs. His justification was that the adjustment of CAGs had caused 'real anguish' and affected public confidence in the exams system, and he pointed to the burden that an appeals process would place on teachers in justifying the decision. |
| **Wales** | The Chief Executive of Qualifications Wales announced on [Monday 29th June](https://qualificationswales.org/english/news/qualifications-wales-publishes-grade-calculation-aims-and-appeals-requirements/) that GCSE and A-Level grades would be awarded via standardised teacher assessment. The aims (consulted upon 'widely') were that students would receive a fair grade, national outcomes would be broadly similar, that no one would be systematically disadvantaged, and a wide range of evidence woul be used to calculate grades. | In an article on the Qualifications Wales website [on 13th August](https://qualificationswales.org/english/news/this-years-qualifications-are-as-valuable-as-any-other-year/), the Chief Executive argued that the method chosen had produced 'meaningful and robust' grades and pointed to 'no statistically significant differences this year relative to other years'. The article pointed out that, following a direction from the Education Minister, no student would receive a lower grade than their corresponding AS-Level grade. | On [Monday 17th August,](https://qualificationswales.org/english/news/statement-on-summer-2020-a-level-as-level-and-gcse-results/) Qualifications Wales announced that - following a subsequent instruction from the Education Minister, learners in Wales would receive their Centre Assessment Grades for GCSEs, A and AS Levels, and the Welsh Baccalaureate Skills Challenge Certificate. Students who had a post-standardisation grade which was higher than their centre assessed grade would be able to use whichever was higher. |
| **Northern Ireland** | [On April 16th,](https://ccea.org.uk/news/2020/april/ccea-announces-how-gcses-and-levels-will-be-awarded-summer-2020) the Northern Ireland qualifications authority CCEA announced that it would award GCSEs, A Levels and AS Levels based on a formula which (a) took school or college assessed grades and (b) applied previous performance data to reach a grade. It announced further information on [August 7th](https://ccea.org.uk/news/2020/august/ccea-publishes-information-standardisation-process-summer-2020-qualifications), with the Chief Executive Justin Edwards highlighting the role that ‘fairness’ played in creating a set of results which were in line with a ‘normal’ year, and that their process was in line with England and Wales. | In announcing GCE grades on the [13th August](https://ccea.org.uk/document/6833), Justin Edwards highlighted the use of similar approaches and methodologies in apportioning grades to England and Wales, describing theirs as the fairest system which would allow students to receive awards and progress to the next stage of study. The standardisation process was presented as ensuring that standards were maintained. On the [11th August](https://ccea.org.uk/news/2020/august/ccea-update-evidence-can-be-used-years-alternative-appeals-process), the CEA had announced further details about the appeals process available to students unhappy with their grades. | [On August 17th](https://ccea.org.uk/news/2020/august/statement-ccea-relation-summer-2020-awarding-levels), the CCEA announced they had been directed by the Education Minister in the Northern Irish Executive that AS and A-Level qualifications would now be awarded on either the higher of the standardised grade, or the grades submitted by schools and colleges. A separate statement [on the same day](https://ccea.org.uk/news/2020/august/ccea-statement-centre-assessment-grades-be-awarded-gcse) announced that GCSEs would be treated the same way. The CCEA announced that they would work quickly to confirm the results and to share the information with UCAS. |

## **Table 2 The algorithms, as described by exams agencies**

| Scotland | Teachers were asked to reach a judgement based on a pupil's year long achievement. The existing grade bands for each subject were subdivided, with teachers asked to (a) place candidates within those new and more narrow bands, and (b) rank their pupils in order. The SQA used these estimates, following a 'checking and validation' process which ensured ‘consistency across schools and colleges and with results from previous years’ ([SQA, 2020](https://www.sqa.org.uk/sqa/93920.html)). The SQA developed a maximum and minimum range of marks for every course at each school based on an average over a four year period. When centres allocated grades outside the accepted range, teaching ranking of pupils were used instead to decide which students should not fall within the grade. ([SQA, 2020](https://www.sqa.org.uk/sqa/files_ccc/SQAAwardingMethodology2020Report.pdf)) |
| --- | --- |
| England | Ofqual's Direct Centre Performance model was based on the historic record of students over three years in each centre (i.e. school or college) in the subject. Adjustments would be made if the class showed better or worse performance overall than its predecessors in GCSE or other assessments. Teachers or lecturers were asked to place students in rank order of expected achievements, and students were slotted into a predetermined range of grades from A* to U. For smaller courses (fewer than 15 students) teacher predicted grades were used without a moderation or standardisation process. ([Ofqual, 2020](https://assets.publishing.service.gov.uk/government/uploads/system/uploads/attachment_data/file/902736/Standardising_grades_in_summerx_2020_-_factsheet_-_20200721_1529.pdf)) |
| Wales | The Banked-unit centre performance (BUCP) approach calculates final grades at A-Level, combining a teacher assessment of final grades with existing A-Level and other completed assessment grades. Grades were calculated by inputting (a) data about prior student attainment, combined with (b) centre assessed grades, and (c) a rank ordering of students by each centre in each subject. It combined data (where available) from AS Level scores to arrive at grades for each centre which were then verified by a senior group for 'validity and comparability', 'reliability' and 'fairness'. ([WJEC, 2020](https://www.wjec.co.uk/media/p0uiqio1/methods-report-qw-regulated-a-levels-1.pdf)) |
| Northern Ireland | Each Centre submitted a Centre Assessed Grade and the rank order of each student within each grade. The ‘Z’ method calculates a grade for each candidate using a z-score, which allows the likely grade distribution in each centre to be predicted. It applies this grade distribution to candidates within each centre to retain their original rank order and to match the predicted grade distribution. The method proposes to: ‘Produce predicted centre grade distributions for A level candidates based on previous AS performance; Build in enhancement for improvements which would have resulted from resits; Grade candidates using the predicted grade distributions and centre rank orders derived from centre assessment grades; Assign grades to candidates within each centre to retain their original rank order and to match the predicted grade distribution’ ([CEA, 2020](https://ccea.org.uk/downloads/docs/ccea-asset/General/Compendium%20of%20approaches%20taken%20and%20formulae%20used%20in%20GCE%20and%20GCSE%20calculation%20of%20grades%2C%20Summer%202020.pdf): 3) |

# Education Minister statements

# Scotland

## Announcing the system

‘Whilst the protection of life is our overriding priority here, we must do our upmost to ensure that the interests and life chances of our young people, due to sit exams from the end of April, are protected. Their achievements must be rightly and fairly recognised. I want the 2020 cohort to hold their heads high and gain the qualifications and awards that they deserve, after many years of hard work. I know they will be very worried by the situation they face and I want them to be assured we are doing all that we can to deliver the best outcome for them. **Scotland’s Chief Examining Officer has advised me that, with the support of the wider education system, a credible certification model can be put in place that can command confidence in the absence of the exam diet** – to ensure that young people in our schools and colleges who, through no fault of their own, are unable to sit exams, are not in any way disadvantaged. **I anticipate that this model will use coursework, teacher assessments of estimated grades and prior attainment as the basis of certification.** In order for such an approach to be effective, the Scottish Qualifications Authority will require relevant units to be completed and coursework and teacher estimates of grades, to be submitted by the agreed deadlines, or indeed sooner where that is possible. We are facing significant disruption at this time, but I appeal to our teachers and practitioners to do all they can - safely - to meet these deadlines and so allow qualifications to be awarded to their young people. So this ask is clear: schools should submit course work and teacher estimates as soon as they can – and by the dates provided. I should stress that these elements form part of the suite of materials provided to the SQA every year when a full exam diet has been in operation.’ - [John Swinney, 19th March 2020](https://www.gov.scot/publications/statement-covid19-managing-impacts-scottish-education/)

## Defending the system

“This year has been exceptionally challenging but these robust processes mean we have upheld standards so that all learners can hold their heads up and move on to the next phase in their life, whether that be further study, employment or training,“ he said. [...] **All exam systems rely on an essential process known as moderation to uphold standards. This ensures an A grade is the same in every part of the country, making the system fair for everyone, and across all years**.” - [John Swinney, 4^th^ August](https://news.gov.scot/news/results-day)

## Announcing the U-Turn

Before I go any further, I want to apologise for that. In speaking directly to the young people affected by the downgrading of awards—the 75,000 pupils whose teacher estimates were higher than their final awards—I want to say this: I am sorry. Sorry as I am, I know that an apology is not enough. I watched the pictures of the spirited, articulate young people demonstrating in George Square on Friday. I have spoken directly to pupils who wrote to me—Nicole Tate, Lauren Steele, Eva Peteranna, Erin Bleakley, Subhan Baig and Eilidh Breslin—and I thank them for the passion and clarity that they brought to our discussions. I have also heard from parents and teachers. I have listened, and the message is clear. They do not just want an apology—they want to see this fixed, and that is exactly what I will now do.

[...] if grades were awarded without moderation, would have represented a very significant increase in the pass rate across the board and a one-year change without precedent in Scottish exam history. To ensure that it carried out what I asked of it, which was that the results were to be certificated on the basis of maintaining standards across all centres, the SQA judged that increases of that nature could not be sustained without moderation.

[...] Despite the headline improvements in the pass rate at national 5, higher and advanced higher, despite the fact that the pass rate among pupils in the most deprived areas increased at a sharper rate than pass rates in the least deprived communities, and despite the fact that there was progress in closing the attainment gap, the results left many young people feeling that their future had been determined by statistical modelling rather than their own capability and capacity. That has left a feeling of unfairness in the minds of young people.

I draw three conclusions from all that. First, we were concerned that grade inflation, through accepting the original estimates from teachers, would run the risk of undermining the value of qualifications in 2020. In the light of events and of listening to young people, we now accept that that concern, which is not without foundation, is outweighed by the concern that young people, particularly from working-class backgrounds, may lose faith in the education system and form the view that, no matter how hard they work, the system is against them. Education is the route out of poverty for young people in deprived communities, and we cannot risk allowing that view to take hold. Secondly, there is a view that relying on teacher judgment this year alone may give young people an incomparable advantage over pupils in other years. That view has to be weighed against the massive disadvantage that Covid has given young people through the loss of schooling, the limited social interaction, the pressure on mental wellbeing and, in some cases, the heartbreak of bereavement. Perhaps our approach to maintaining standards for the 2020 cohort alongside every other year—even though 2020 is so unique—did not fully understand the trauma of Covid for that year group and did not appreciate that a different approach might help to even things out. Thirdly, this year, 2020, is and must be seen as unique. It has turned our society upside down. It cannot fairly be compared with previous years, and it cannot set an automatic precedent for future years. However, it perhaps merits taking a different approach in relation to certification.

[...] I can confirm to Parliament that all downgraded awards will be withdrawn. Using powers that are available to me in the Education (Scotland) Act 1996, I am today directing the SQA to reissue those awards based solely on teacher or lecturer judgment. Schools will be able to confirm the estimates that they provided for pupils to those who are returning to school this week and next week. The SQA will issue fresh certificates to affected candidates as soon as possible and—this is important—will inform the Universities and Colleges Admissions Service and other admission bodies of the new grades as soon as practical in the coming days to allow for applications to college and university to be progressed. - [John Swinney, Scottish Parliament statement announcing U-Turn, 11^th^ August](https://www.theyworkforyou.com/sp/?id=2020-08-11.7.0&s=speaker%3A14002#g7.6)

# England

## Announcing the system

I recognise that what schools will be doing in these circumstances will look very different from the normal state of affairs, and will ensure that leaders have the flexibility that they need to face this challenge. In order to allow schools and other settings to focus on this new operational model and the support they can give to these young people, we are removing various duties. Ofsted has ceased all routine inspection of early years, schools, colleges and children’s social care services. I can confirm that we will not go ahead with assessments or exams, and that we will not be publishing performance tables for this academic year. We will work with the sector and Ofqual to ensure that children get the qualifications that they need. My Department is working closely with local authorities, representatives of early years, schools and headteachers, regional school commissioners and bodies such as Ofsted and Ofqual about how to deliver this change as effectively as possible. We will do whatever is necessary to support local authorities, schools and teachers through the weeks and months ahead. - [Gavin Williamson, House of Commons](https://www.theyworkforyou.com/debates/?id=2020-03-18a.1083.0&s=speaker%3A24729#g1083.1), 18^th^ March

## Announcing further details of system

As I announced to the House on 18 March, in light of the unprecedented public health emergency the Government has taken the difficult decision to cancel all exams due to take place in schools and colleges in England this summer. We recognise that students have been working hard towards these exams, and this is not a decision we have taken lightly.

Our priority is to ensure that students can move on as planned to the next stage of their lives, including starting university, college or sixth form, or an apprenticeship or a job in the autumn. For GCSE, A and AS level students we will also make sure they are awarded a grade which reflects their work. Our intention is that a grade will be awarded this summer, based on the best available evidence, including any non-exam assessment that students have already completed. There will also be an option, for students who do not feel this grade reflects their performance, to sit an exam at the earliest reasonable opportunity once schools are open again.

**Ofqual will develop and set out a process that will provide a calculated grade to each student which reflects their performance as fairly as possible, and will work with the exam boards to ensure this is consistently applied for all students.** The exam boards will be asking teachers, who know their students well, to submit their judgement about the grade that they believe the student would have received if exams had gone ahead. **To produce this, teachers will take into account a range of evidence and data including performance on mock exams and non-exam assessment – clear guidance on how to do this fairly and robustly this will be provided to schools and colleges. The exam boards will then combine this information with other relevant data, including prior attainment, and use this information to produce a calculated grade for each student, which will be a best assessment of the work they have put in.**

Ofqual and exam boards will be discussing with teachers’ representatives before finalising an approach, to ensure that it is as fair as possible. More information will be provided as soon as possible. The aim is to provide these calculated grades to students before the end of July. In terms of a permanent record, the grades will be indistinguishable from those provided in other years. We will also aim to ensure that the distribution of grades follows a similar pattern to that in other years, so that this year’s students do not face a systematic disadvantage as a consequence of these extraordinary circumstances. Furthermore, university representatives have confirmed that they expect universities to be flexible and do all they can to support students and ensure they can progress to higher education.

We recognise that some students may nevertheless feel disappointed that they haven’t been able to sit their exams. If they do not believe the correct process has been followed in their case, they will be able to appeal on that basis. In addition, if they do not feel their calculated grade reflects their performance, they will have the opportunity to sit an exam at the earliest reasonable opportunity once schools are open again. Students will also have the option to sit their exams in summer 2021.

There is a very wide range of different vocational and technical qualifications as well as other academic qualifications for which students were expecting to sit exams this summer. These are offered by a large number of awarding organisations and have differing assessment approaches – in many cases students will already have completed modules or non-exam assessment which could provide evidence to award a grade. We are encouraging these organisations to show the maximum possible flexibility and pragmatism to ensure students are not disadvantaged. Ofqual is working urgently with the sector to explore options, and we will work with them to provide more details shortly.

The Government will not publish any school or college level educational performance data based on tests, assessments or exams for 2020. - [Gavin Williamson, Written Ministerial Statement](https://www.theyworkforyou.com/wms/?id=2020-03-23.HCWS176.h&s=speaker%3A24729#gHCWS176.0), 23^rd^ March

<https://www.gov.uk/government/news/ofqual-gcse-and-a-level-consultation-outcomes-and-autumn-exam-series-proposals> Ofqual

## Tweaking the system

“Every young person waiting for their results wants to know they have been treated fairly. By ensuring students have the safety net of their mock results, as well as the chance of sitting autumn exams, we are creating a triple lock process to ensure confidence and fairness in the system. No one wanted to cancel exams – they are the best form of assessment, but the disruption caused by Covid-19 meant they were not possible. This triple lock system will help provide reassurance to students and ensure they are able to progress with the next stage of their lives.” Williamson, 12^th^ August

## Defending the system

"The system, for the overwhelming majority of young people, is going to deliver, you know, credible, strong results for every single one of them. "It's a robust system, it's a fair system, it's making sure that young people get the grades that they've worked so hard towards." He also defended the last-minute "triple lock", saying: "I'm not going to hesitate in terms of actually making changes if I can get the system as fair as possible for every single child." - [Gavin Williamson, BBC News interview, 12^th^ August](https://www.bbc.co.uk/news/education-53750864)

## More resources

Ofqual defence of system <https://www.gov.uk/government/news/response-to-the-secretary-of-states-announcement-of-12-august-2020>

House of Commons Education Committee response <https://www.theguardian.com/education/2020/jul/10/mps-warn-bias-predicted-results-pupils-england>

Useful timeline of events <https://www.theguardian.com/education/2020/aug/17/uk-exams-debacle-how-did-results-end-up-chaos>

## Announcing the U-Turn

“We worked with Ofqual to construct the fairest possible model, but it is clear that the process of allocating grades has resulted in more significant inconsistencies than can be resolved through an appeals process,” Williamson said, in remarks released by his department. [...] We now believe it is better to offer young people and parents certainty by moving to teacher assessed grades for both A- and AS level and GCSE results. I am sorry for the distress this has caused young people and their parents, but hope this announcement will now provide the certainty and reassurance they deserve.” - [Gavin Williamson statement](https://www.theguardian.com/education/2020/aug/17/a-levels-gcse-results-england-based-teacher-assessments-government-u-turn), 17^th^ August

# Wales

## Announcing the system

We are in an unprecedented period, one that is changing hour-by-hour, and governments around the world are having to make quick decisions. We recognise the worry and anxiety that the uncertainty around the summer exam series was causing. Today I met with Qualifications Wales and WJEC to consider options that are in the best interests of our learners. We recognise that there are no easy choices but we have agreed that the best way forward is not to proceed with summer exam series. Learners due to sit their GCSEs and A levels this summer will be awarded a fair grade to recognise their work, drawing on the range of information that is available. We will be working with the sector to announce further details shortly but wanted to give this early certainty. We also won’t be using the results to publish performance measure outcomes in 2020. - [Kirsty Williams](https://gov.wales/written-statement-written-statement-summer-examinations-2020), 18^th^ March

Details about years 10 and 12 <https://gov.wales/education-minister-announces-exam-arrangements-years-10-and-12>

## Further announcement

I am pleased to confirm that the 2020 Wales results day for AS and A level students, and for GCSE students, will be as originally scheduled. This means that Wales, Northern Ireland and England will share the results days of 13 August for AS & A Level and 20th August for GCSE. As I set out in my letter to the Education Secretary for England on 31 March, during these uncertain times, providing clarity and ensuring that learners are not disadvantaged in accessing their university places by a divergence in results dates have been key priorities for me. I am pleased this will now be the case. Sticking to the existing results dates has been the intention in Wales, supported by the advice of our independent exam regulator Qualifications Wales. **Proposals in England to move to a much earlier results day introduced an unnecessary risk, and I had expressed my concerns to counterparts in the UK government, as had the education sector here in Wales and UCAS**. I therefore welcome today’s announcement for England. Education ministers from across all governments in the UK have worked well together during these challenging times, but on this occasion I regret that we were unable to make a joint announcement. GCSEs and A Levels are shared and owned across three countries within the UK. Making a joint announcement would have helped provide much needed assurance and certainty for all of our learners, their parents and practitioners at this difficult time. I hope that now confirmation of results day has been provided our learners are able to make future plans with a little more confidence, although I recognise this continues to be a time of great uncertainty. – [Kirsty Williams, 16^th^ April](https://gov.wales/written-statement-results-day-2020) [[Qualification Wales description of system](https://gov.wales/sites/default/files/publications/2020-09/ATISN%2014261%20-%20Doc%202.pdf)*]*

## Defending and tweaking the system

"Students in Wales, and prospective employers and universities across the UK, can be assured that their A-level grades reflect their work and externally assessed exams," she said. [...] Almost half the final grade comes from AS-level exams - this is not the situation elsewhere. [...] Therefore, in building on that completed work, I am giving a guarantee that a learner's final A-level grade cannot be lower than their AS-grade.

If a student receives a final grade tomorrow that is below that of their previous AS grade, then a revised grade will be issued automatically by WJEC." - [Kirsty Williams, BBC News, 12th August](https://www.bbc.co.uk/news/uk-wales-politics-53753118)

“I want to send my very best wishes to everyone who receives their A-level, AS, Welsh Bacc and vocational qualification grades today. “Due to the many changes we’ve had to make this year in exceptional circumstances, you’ve had to make many sacrifices. “But you have every reason to be proud of all the work you’ve done, which will serve you well, and proud of the determination you have shown to overcome this challenging time. “As announced yesterday, we have guaranteed that a learner’s final A Level grade cannot be lower than their AS grade. Students in Wales, and prospective employers and universities across the UK, can be assured that their A Level grades reflect their work and externally assessed exams. “I hope you get the grades you’d hoped for, and you can continue with your educational journey in the autumn. Although many of you will be pleased with your results and excited for your next step, if you didn’t get what you’d hoped, there’s plenty of options & advice on Working Wales. “Best of luck, and best wishes for the future.” - [Kirsty Williams statement on exam day (13th August)](https://www.abergavennychronicle.com/article.cfm?id=117670&headline=Statement%20from%20Education%20Minister,%20Kirsty%20Williams%20on%20today%27s%20exam%20results&sectionIs=News&searchyear=2020)

## Announcing the U-Turn

"Recent months have thrown up unexpected, new and complex challenges [...]. Working with Qualifications Wales and the WJEC we looked for an approach which provided fairness and balanced out differences in the standards applied to judgments in schools. But, as I announced yesterday, and given decisions elsewhere, the balance of fairness now lies with awarding centre assessment grades to students." - [Kirsty Williams, 18th August](https://www.bbc.co.uk/news/uk-wales-53817855)

# Northern Ireland

## Announcing the system

“In March, I announced that, in line with other jurisdictions of the UK, GCSE, AS and A level exams would not proceed in Northern Ireland in summer 2020. “My priority is to ensure that pupils receive fair results that reflect their hard work and enable judgements to be made about their future progression to study or employment or other avenues. CCEA, the local examinations body, together with officials from my departments and others in the education sector have been working tirelessly to develop an alternative process. This is a very complex process and there is still more work to be completed, however, it is vital that pupils and their families receive some clarity on this issue. Consultation on the preferred options has been carried out with key education stakeholders, including head teachers’ representatives and teaching unions.

Today, I have set out how qualifications will be awarded for GCSE and A level pupils. **This will involve teachers using their professional judgement together with an extensive range of evidence held by schools, and statistical modelling. There will also be a process for standardising outcomes across school and other examination centres.** The development of an appropriate appeals mechanism is also under consideration as well as arrangements for private candidates. Whilst there will not be an opportunity for pupils to take re-sits in the autumn, they can, of course, choose to re-sit in the summer of 2021. I will also seek to align the issuing of results with England and Wales (ie A level results on 13 August and GCSEs on 20 August), this ensuring that local candidates are not put at a disadvantage. I have instructed CCEA to take forward work to implement these decisions, and they will issue more detailed guidance to schools, pupils and parents as a matter of urgency. My officials have consulted with key education stakeholders, including head teachers’ representatives and teaching unions, on the preferred options presented by CCEA. In reaching my decisions I have taken account of the views expressed through these consultations. I am reassured that all parties recognise that there is no perfect solution, but agree that we have identified the most appropriate available solutions given the unprecedented circumstances.” - [Peter Weir, Northern Ireland Education Minister, 16th April](https://www.education-ni.gov.uk/news/minister-announces-arrangements-summer-examinations)

## Defending the system

It would not only be unfair between pupils in different years, but it would be unfair within years, because you would get some teachers who would be quite tough in their assessment and others who would be very, very generous. There would be no equality whatsoever. It would depend, from teacher to teacher, from centre to centre, on what attitude and approach that they took. That would mean that some pupils would get a large boost beyond what they have got at present. Sometimes that might be fair; sometimes it would not be fair. Others would actually be held back and their grade would not be changed. It would not create a level playing field between students. Yes, you can take the individual bits, but if you simply apply that blanket approach, you will lack any credibility in the examination qualifications. It would take us, for example, out of sync with England, which is about 85% of the UK market. As a small region, more so than anywhere else, if we damage our credibility and it is felt that the grades from Northern Ireland examinations are not really worth the paper that they are written on, that will be of massive detriment: "Oh, that grade is from Northern Ireland. That is not as good as a grade from somewhere else." It would also create an inequality for the students who, for instance, take examinations from examination boards outside Northern Ireland, so we would not even have equality within Northern Ireland. - [Peter Weir, 14th August 2020](http://aims.niassembly.gov.uk/officialreport/minutesofevidencereport.aspx?AgendaId=23097&eveID=12139)

## Announcing the U-Turn

I have today instructed CCEA that all of their AS and A level qualifications will now be awarded the higher of the grade submitted by their centre or the grade calculated by CCEA. CCEA is working to release the revised results to candidates as quickly as possible. In the interim, students will be able to access details of their Centre Assessed Grade from their school or college. “Concerns remain over the impact of changes to the qualifications system throughout the United Kingdom and any potential solution offered has its flaws. However, my prime concern is to ensure that young people in Northern Ireland are in no way disadvantaged in comparison to their peers elsewhere. Portability and comparability of qualifications is critical for students, particularly in Northern Ireland.

Whilst standardisation is normally an important feature of awarding qualifications, these are truly unique circumstances and this approach is now being adopted across the UK. This is why I have taken this decision today. In the challenging situation in which we find ourselves, there are no perfect solutions. Students would have preferred to have taken their exams, but that was simply not possible in the circumstances. There is no substitute for exams themselves and recent events highlight the need for a full-time return to education, five days a week. I want to thank our teachers and school leaders for all their hard work during this extremely challenging time. Our young people have shown their tremendous attitude and resilience though the Covid-19 pandemic. I hope today’s decision will provide a platform for them to go forward positively into higher education, employment or training. – [Peter Weir, 17^th^](https://www.education-ni.gov.uk/news/changes-and-level-awards-northern-ireland) August

# Early developments for exams in 2021

Each government announced an additional year of exams replacement, but with limited discussion of the details. Policymakers were aware of the unequal impact of temporary school closures, but took time to act consistently.

- On 7^th^ October 2020, the Scottish Government cancelled the 2021 National 5 exams as they represented ‘too big a risk’ in the context of the pandemic ([Holyrood Magazine, 2020](https://www.holyrood.com/news/view,national-5-exams-to-be-cancelled-in-2021#:~:text=National%205%20level%20exams%20are,by%20the%20COVID%2D19%20pandemic.)), but insisted that rescheduled Higher and Advanced Higher exams would go ahead ([BBC News, 2020](https://www.bbc.co.uk/news/uk-scotland-54423265)). It then cancelled the latter on 8^th^ December, with Swinney arguing that ‘exams cannot account for differential loss of learning and could lead to unfair results for our poorest pupils’ ([RTE, 2020](https://www.rte.ie/news/uk/2020/1208/1183185-scotland-exams/#:~:text=All%20exams%20in%20Scotland%20have,Secretary%20John%20Swinney%20has%20announced.&text=%22This%20could%20lead%20to%20pupils,That%20is%20simply%20not%20fair.%22)).
- The Welsh Government announced on 10^th^ November that all exams for the forthcoming year would be cancelled ([Welsh Government, 2020](https://gov.wales/wales-approach-qualifications-2021-confirmed-education-minister-kirsty-williams)).
- The UK Government continued to insist that exams would take place, but announced their cancellation on 8^th^ December. The UK Government announced the cancellation of the summer exam diet on 6^th^ January 2021, with Williamson citing the need to close schools as his motivation.
- Weir made the same announcement for Northern Ireland on the 6^th^ January 2021 ([ITV News, 2020](https://www.itv.com/news/utv/2021-01-06/education-minister-set-to-cancel-gcse-as-and-a2-exams)).

At the time of writing (January 2021), the plans for awarding grades in lieu of are unpublished. However, some guiding principles can be observed.

- Swinney stated that the Scottish Government and SQA ‘will adopt the new model that has been developed and base awards on teacher judgement of evidence of learner attainment’ ([Holyrood, 2020](https://www.holyrood.com/news/view,national-5-exams-to-be-cancelled-in-2021#:~:text=National%205%20level%20exams%20are,by%20the%20COVID%2D19%20pandemic.))
- Williamson stated that the awards to English GCSE and A-Level students will not be algorithm based ([Sky News, 2020](https://news.sky.com/story/covid-19-gcse-and-a-level-exams-to-be-replaced-by-teacher-assessments-in-england-this-summer-12180579)).
- Williams stated that a system of teacher assessment is being developed which would ensure that 'centre based outcomes which will be linked to an agreed national approach to provide consistency across Wales' ([Welsh Government, 2020](https://gov.wales/wales-approach-qualifications-2021-confirmed-education-minister-kirsty-williams)).
- Weir stated on ITV News on January 6^th^ that his Education Department were “working with CCA to make sure that contingency plans will be developed that will be revealed before the end of the month".
